# Bone remodeling markers: monitoring the changing bone

**DOI:** 10.1515/almed-2024-0009

**Published:** 2024-02-27

**Authors:** Nerea Varo

**Affiliations:** Clínica Universidad de Navarra – Bioquímica, Pamplona, Spain

Transition to bipedalism was crucial to human evolution and is a distinctive characteristic of human beings that differentiates us from our primate ancestors. Walking on two legs definitely gave us a variety of advantages. However, the human skeleton underwent substantial morphological adaptations, including an S-shaped spine curvature and caused changes to the pelvis for the skeleton to be able to bear the burden.

The skeleton is a highly-specialized tissue that undergoes continuous remodeling throughout life. Around 20 % of bone tissue is replaced annually by the remodeling process. This process is mediated by coordinated action of osteoclasts and osteoblasts. When a disbalance occurs between these processes, bones turn more porous. Osteoporosis was first described by the French anatomist Joseph Guichard Duverney (1648–1730) in his posthumous book *Treatise on bone diseases*. The term “osteoporosis” was first coined in 1833 by the French physician Lobstein to refer to porous bones observed in autopsies. Currently, osteoporosis is defined as “a skeletal disease characterized by a reduced bone strength, which increases the risk for fractures”. Therefore, osteoporosis is established as a function of bone strength, which is determined by the combination of bone density and bone quality.

Osteoporosis is the most common metabolic bone disease. Determining the prevalence of this disease is challenging, as it remains asymptomatic until fractures occur. For this reason, osteoporosis is called “the silent epidemic”. The last report of the International Osteoporosis Foundation (IOF) reveals that 25.5 million women and 6.5 million men had osteoporosis in the European Union, Switzerland, and United Kingdom in 2019. Additionally, 4.3 million new fractures were associated with bone frailty [1]. In our country, prevalence is estimated to be three million people, of which near 80 % are women. Fractures related to bone frailty are the direct result of osteoporosis. In addition, fractures are responsible for a significant reduction of quality of life, have a high social cost for the dependence they cause, and are associated with a high economic cost. Indeed, in Spain, the direct cost of osteoporotic fractures was 1,813 million euros and the economic burden of the disease accounted for 3.8 % of healthcare expense.

The diagnosis of osteoporosis is established in accordance with the 1994 WHO criteria, on the basis of bone mineral density, expressed as the number of standard deviations of a subject’s bone mineral density above or below the mean bone density of the young adult population of the same sex [2]. However, the use of bone densitometry as the only diagnostic and follow-up tool available for osteoporosis is not exempt from some limitations. Some of these limitations include that densitometry provides quantitative, but not qualitative information, has a low predictive value for fractures and a low sensitivity. Finally, it takes time to reflect small changes in bone mineral density.

Other options have been explored, such as bone remodeling markers, which are products released to the bloodstream during bone formation and resorption. Hence, these markers can be used to evaluate these two processes. Traditionally, they are classified as bone formation and bone resorption markers. Alkaline phosphatase was the first of these markers to be included in clinical practice (1929). The method used for measuring alkaline phosphatase was first described by King & Armstrong, modified by Ohmori, Bessey, Lowry & Brock, and finally optimized by Hausamen et al. The techniques currently used in clinical laboratories are based on modifications to these methods.

In the early 80’s, metabolic bone diseases drew the attention of clinicians and more specific assays were developed. Osteocalcin was one of the first bone markers developed and described to indicate osteoid formation, measured by bone biopsy. The 90’s was a decade of a major expansion in the development of new bone-specific assays. Thus, assays based on antibodies against the N-mid region of osteocalcin were developed. Other novel assays included radioimmunoassays for bone alkaline phosphatase, with very-low reactivity against the hepatic isoform, which were used to identify patients responsive to alendronate. Some time later, the methods for cross-linking pyridinoline, pyridinoline and deoxypyridinoline in urine enabled clinicians to find that increased clearance was associated with the rate of postmenopausal bone loss. Automated methods emerged to measure C-terminal telopeptide of type I collagen (CTX) and N-terminal propeptide of procollagen type I (PINP) in serum by immunoassay.

The enthusiasm raised by the promising characteristics of bone remodeling markers decreased due to the heterogeneity and preanalytical variability of the assays available. These limitations hindered the spread of these methods in clinical practice. The IOF/IFCC joint Working Group on Bone Marker Standards (WG-BMS) recommended using PINP and CTX in blood as the bone markers of reference in observational and interventional studies, as well as for monitoring antiosteoporotic treatment [3]. The WG-BMS emphasized the need to standardize or harmonize commercially available assays to generate comparable data that could be used in the clinic and in research. The IFCC Committee on Bone Metabolism (IFCC C-BM) is preparing international reference materials. At clinical level, the IFCC is collaborating with the IOF to establish reference universally-accepted intervals and cut-offs. In this special issue, the factors influencing marker variability will be reviewed.

Regarding its clinical use, the most important guidelines, the RACGP (Royal Australian College of General Practitioners) the ESCEO-IOF (European Society for Clinical and Economic Aspects of Osteoporosis-International Osteoporosis Foundation); the NOGG (National Osteoporosis Guideline Group); (North American Menopause Society); the ES (Endocrine Society); and the ACOG (American College of Obstetricians and Gynecologists) agree that the use of bone markers should be limited to treatment monitoring purposes and solely in specialized centers. In our country, consistently with international organizations, the SEIOMM (2022) pointed out that diagnosis of osteoporosis does not necessarily require measuring bone remodeling markers. However, these markers are recognized to be useful to assess therapeutic response when measured in standard conditions.

To reduce preanalytical variability, the National Bone Health Alliance (NBHA) published some recommendations for laboratories, reagent manufacturers, and clinicians about patient preparation and sample handling [4]. Controllable and incontrollable patient-related factors were reviewed to facilitate interpretation and sample collection. Samples for CTX measurement must be collected in the morning, with the patient in fasting conditions due to its circadian variability. In addition, EDTA plasma is recommended to ensure sample stability. The conditions for PINP sample collection are less strict, as this marker has minimal circadian variability and is not affected by food intake. Certainly, the adoption of standard procedures for sample handling and preparation reduces controllable preanalytical variability significantly. Non-controllable variables (age, sex, pregnancy, immobilization, recent fracture, comorbidities, antiosteoporotic drugs or other medications) should also be taken into account in the interpretation of bone remodeling marker results. Successful compliance with these recommendations requires close collaboration of the stakeholders involved, including laboratories, the medical community, and reagent manufacturers.

This monographic issue addresses the challenges faced by laboratory specialists. A summary is provided of factors associated with changes in bone remodeling markers. Focus is also placed on the role of the bone as an organ that induces the production of regulatory molecules in other tissues. Ostheoporotic fracture is a non-classical complication of diabetes that is frequently overseen, possibly due to its complex diagnosis and management. An overview is provided of the effects of antidiabetic drugs on bone metabolism, added to the association between osteocalcin levels and bone mineral density and vitamin D-receptor gene polymorphisms in diabetes type 1 and 2. Finally a study is presented on bone metabolism in children with overweight/obesity.

In the light that osteoporosis and frailty fractures are frequent and can be effectively treated, most patients are preferably treated in primary care settings, with complex cases being referred to specialty care. In Spain and other countries, there is not a medical specialty for the management of bone disease. Hence, patients are treated by different specialists, according to the Autonomous Community, including internal medicine specialists, traumatologists, gynecologists, endocrinologists and rheumatologists. The management of bone disease requires specific training and education. Consistency and standard patient care is limited by the variability of the specialists that treat bone diseases. Due to their transversal nature, clinical laboratories have a privileged position to promote standardized patient care, protocols, preanalytical phase procedures, and standard test request procedures. Hence, standardization will facilitate the interpretation of bone markers results. Laboratory specialists should assume these roles with rigor by adopting a scientific approach. In addition, the large amount of results obtained in daily practice in all laboratories may be exploited to generate scientific evidence, develop hypotheses, and plan future interventional studies, as suggested by ESCEO.

Our current lifestyle will lead to adaptations in our skeleton from a still-uncertain evolutionary approach. In the meanwhile, successful management of these challenges will relief the burden of public health systems and benefit clinicians and patients at high risk for fractures.
